# An Aged Primipara

**Published:** 1868-08

**Authors:** 


An Aged Primipara.—In response to the inquiry made through the *London Lancet* with respect to child-bearing in advanced life, Dr. Cachot, of St. Mary's Hospital, informs us that he delivered, in that institution, a female of her first child, at the age of 53 years, and again in sixteen months. The labor in both confinements was tedious, from inertia of the uterus, and required the forceps. The mammary glands enlarged but produced no milk. The children lived in hoth cases.

